# Supplementary material for: Using mobile phone data to estimate dynamic population changes and improve the understanding of a pandemic: A case study in Andorra
Source: PLoS One. 2022 Apr 26;17(4):e0264860. doi: 10.1371/journal.pone.0264860 (PMC9041757; doi:10.1371/journal.pone.0264860)
Supplement: S1 Appendix — (PDF) [file pone.0264860.s001.pdf]

S1 Appendix

A.1 Andorra and COVID-19

March 2	First COVID-19 case confirmed in Andorra.
March 12	Ski stations closed.
March 13	Partial confinement: school closures and recommended confinement.
March 18	Total confinement: all non-essential activities ordered closed.
April 7	Beginning of masks delivery and progressive use by population.
April 17	Allowance to 1 hour of walk in 1km radius every two days.
April 20	Phase 1 reopening: low risk economic activities resume; 1000 people return to work.
May 4	Start of 1st round of population serology screening.
May 4	Phase 2 reopening. Additional 4760 workers return to normal activity.
May 13	Increase to 2 hours of activity every day to walk or exercise.
May 18	Start of 2nd round of population serology screening.
May 18	Phase 3 reopening: additional 3300 workers returned to normal activity.
June 1	Confinement restrictions completely lifted.
June 15	French and Spanish borders opened (with restrictions on Spanish side).
June 21	Spain ended state of emergency and further lifted border controls.
July 1	Remaining Spanish border restrictions lifted.
September 1	Massive testing for teachers and school kids began.

Table A.1. Timeline of the COVID-19 related dates in Andorra.

A.2 Home parish inference

The parish-level populations inferred from the telecoms data for May 2020 were compared to published 2020 population statistics [1]. There is a Pearson correlation coefficient of 0.959 ($p<0.001$), suggesting that the telecoms data are representative of the true population.

Parish	Published population	Inferred mobile subscriber population
Andorra la Vella	22504	13555
Canillo	4371	4367
Encamp	11716	6766
Escaldes-Engordany	14626	7366
La Massana	10199	6165
Ordino	4957	2427
Sant Julià de Lòria	9374	4339

Table A.2. Published parish populations compared to parish populations inferred from telecoms data.

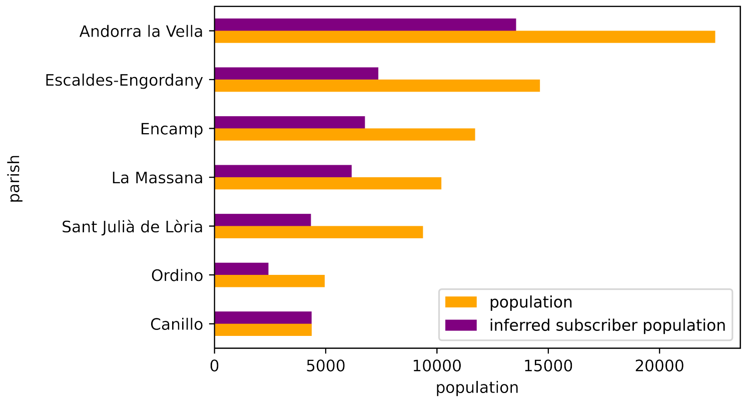

Fig A.1. Published parish populations compared to parish populations inferred from telecoms data There is a Pearson correlation coefficient of 0.959 ($p<0.001$), suggesting that the data are representative of the true population.

A.3 Comparing entrances and infection rates between Andorra, France, and Spain

The SEIR model (iii) in this work incorporates trips and entrances data to model transmission rates. It includes an assumption that the likelihood of new country entrants being infectious tracks with the timeline of infection rates in Andorra. In order to check this assumption, we compare the rates of COVID-19 between the country of Andorra and its bordering neighbors, Spain and France, during our period of study. This is done by comparing the timeline of reported deaths per 1 million residents, where data is smoothed over a 7 day rolling window. Death reports are used instead of case reports as a more stable comparison indicator in this study and others because death reports were considered to be less impacted than case reports by the dynamically changing testing procedures which varied by country [2,3]. The Pearson correlation coefficients between Andorra and France and Andorra and Spain are 0.916 ($p < 0.001$) and 0.928 ($p < 0.001$), respectively. We note there was an error in the Spain data with negative deaths values in late May. We changed the negative values to 0 for this comparison. Without the change, the correlation was still statistically significant with Pearson correlation coefficient 0.918 ($p < 0.001$). The timeline of infections is shown in Fig A.2.

During this same period, telecoms data showed that 86% of entrances by foreign SIMs were either Spanish or French, and 68% of all entrances were by Spanish or French SIMs when accounting for entrances by Andorran SIMs. The timeline of entrances by SIM nationality is shown in Fig A.3.

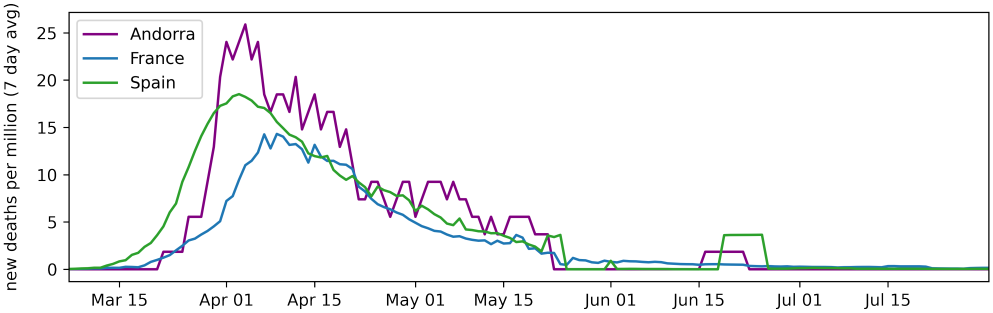

Fig A.2. Comparison of reported deaths per 1 million residents between the country of Andorra and its only bordering neighbor countries, Spain and France. The Pearson correlation coefficients between Andorra and France and Andorra and Spain are 0.916 ($p<0.001$) and 0.928 ($p<0.001$), respectively.

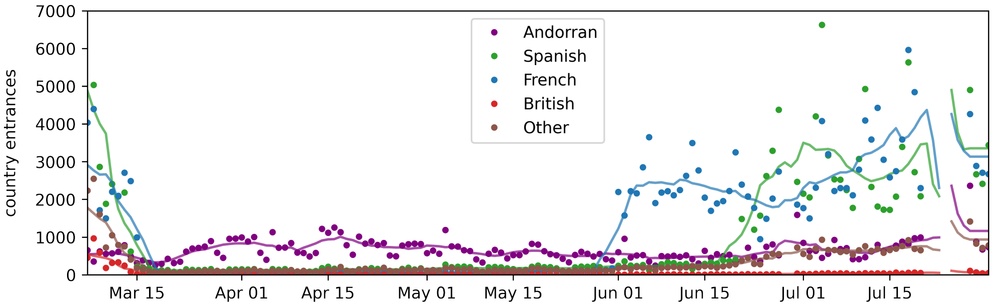

Fig A.3. The timeline of country entrances, measured via Andorra Telecom data, by mobile subscribers' SIM nationality. During the period of March through July, 2020, 86% of entrances by foreign SIMs were either Spanish or French, and 68% of all entrances were by Spanish or French SIMs when accounting for entrances by Andorran SIMs.

A.4 Serology tests and country departures

Note that the sum of survey participation varies due to missing values regarding the participants' parish of residence or temporary worker status.

	survey 1 participants (N)	survey 1 seroprevalence	survey 2 participation
all	70,626	9.7%	91.6%
temporary workers	2,714	13.3%	70.7%

Table A.3. Serological survey participation and results for temporary workers versus general population. Seroprevalence results were previously reported by Royo-Cebrecos et al. Survey 2 participation indicates how many individuals who participated in survey 1 also participated in survey 2.

Two cross sectional serological surveys were conducted in Andorra from May 4-28, 2020, using a rapid serological test (nCOV IgG/IgM) [4]. For each participant, the test data include the dates of their participation in surveys 1 and/or 2, the positive versus negative results for IgG/IgM antibodies for each round of testing, the participant's parish of residence, whether the participant is a temporary worker, and other demographic information. Table A.3 shows the number of participants and seroprevalence from survey 1 as well how many participants from survey 1 also participated in survey 2. Data for temporary workers is highlighted. Seroprevalence data is from previously reported results and was calculated based on the number of individuals who had a positive result of IgG and/or IgM [4]. The testing was voluntary; an issue with the testing was that many people who participated in the first survey did not participate in the second survey. Seroprevalence was higher among temporary workers. At the same time, temporary workers who participated in survey 1 were less likely to participate in survey 2 versus the general population. See Table A.3.

parish	serology survey participants			2020 mobile subscribers present			2019 mobile subscribers present		
	survey 1 (N)	survey 1 & 2	**decline**	survey period 1	survey periods 1 & 2	**decline**	survey period 1	survey periods 1 & 2	**decline**
Andorra la Vella	4542	4262	**6.2%**	15803	15415	**2.5%**	27531	23295	**15.4%**
Canillo	4495	4017	**10.6%**	5406	5121	**5.3%**	5095	3936	**22.7%**
Encamp	10846	10273	**5.3%**	7613	7449	**2.2%**	8102	7397	**8.7%**
Escaldes-Engordany	28924	27688	**4.3%**	8336	8172	**2.0%**	9089	6961	**23.4%**
La Massana	8843	8451	**4.4%**	7389	7214	**2.4%**	9813	8365	**14.8%**
Ordino	4214	4049	**3.9%**	2821	2758	**2.2%**	3446	2964	**14.0%**
Sant Julià de Lòria	8632	8295	**3.9%**	5056	4929	**2.5%**	9201	8197	**10.9%**

Table A.4. Serological survey participation and mobile subscribers by home parish.

We counted the number of mobile subscribers, by inferred home parish, who were in the country during the first and second survey periods (May 4-14 and May 18-28, 2020). Subscribers were counted as present during a survey period if they had at least one "stay" within the period. We estimated the rate at which subscribers left the country after the first test by counting how many subscribers were present during only the first period versus both periods.

These numbers were compared to the parish-level serology test participant populations. Namely, the portion of serology survey participants who did survey 1 but not survey 2 was compared to the estimated portion of mobile subscribers who left the country between survey periods. There is a statistically significant Pearson correlation coefficient of 0.937 (p=0.0019).

To further validate that the decline in test participation was related to people leaving the country, we repeated these tests using 2019 telecoms data. In this case, there is a Pearson correlation coefficient of 0.4928 (p=0.2612), which is not statistically

significant. If the May 2020 subscribers had left the country for reasons not related to the pandemic, we would expect the correlation to be similar for the 2019 and 2020 data.

A.5 Model training details

All modeling work is publicly accessible in a Python Jupyter notebook: `https://github.com/CityScope/CSL_Andorra_COVID_Public/blob/main/analysis/SEIR_models_trips_entrances.ipynb`.

Optimal parameters were estimated by minimizing the negative log-likelihood function using the L-BFGS-B method with the Python SciPy library [5, 6]. This step searches for optimal parameters by taking initial parameters which are then modified towards improved values, with specified bounds. The (minimum, maximum) bounds for γ were set to (1/10, 1/2). The bounds used for each of the parameters related to β - $b0, b1, b2, b3, b4$ - were (0, 2), with the observation that the parameters for the best fit models did not come up against these bounds. The (minimum, maximum) bounds for both $E(0)$ and $I(0)$ were set to (40, 4000), where t=0 corresponds to March 14, the first day of the training period. The values of (40, 4000) were conservatively set where the minimum was based on reported active cases, and the maximum based on reported cumulative cases. In addition, the training routine discarded models where E(0) and I(0) values differed by more than 3000.

Before describing how initial parameters were handled, first note that the optimizing function is not convex. To avoid the optimization function terminating at local minima, a grid search was used for the initial parameters. The same grid search method was used for each of the models.

In addition to the grid search routine, another step was taken to find optimal model fits: The models using the trips and entrances data were initially fit using spline approximations of these metrics, where the splines were estimated from the true metrics using knots spaced by 7 days. Fig A.4 shows the comparison of the true metrics versus their spline approximations. This step was taken to further smooth the data and ease the computational complexity of the model fitting routine. Without this improvement, the model training was slow and rarely resulted in successful outcomes, and the estimated parameters rarely varied from their initial values.

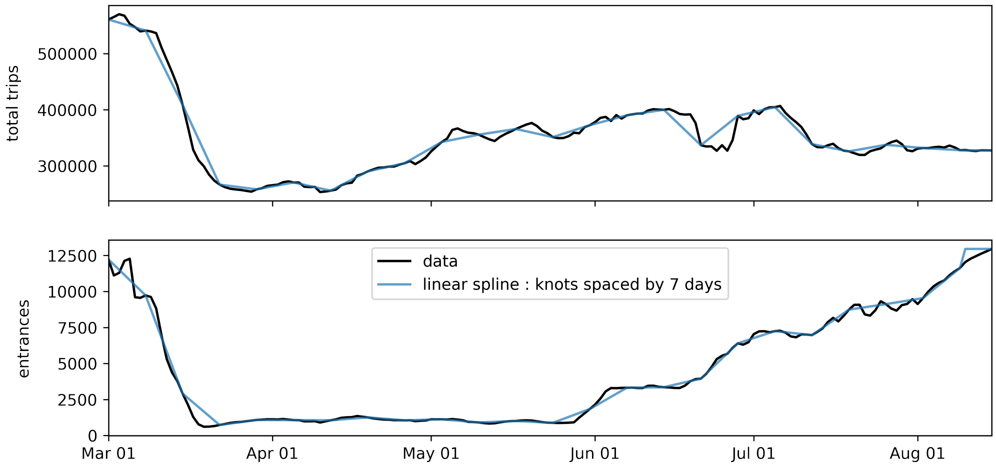

Fig A.4. Metrics and spline approximations. Daily estimated total trips and entrances metrics, and the linear spline approximations of these metrics where knots are spaced by 7 days.

After the models were fit using the spline approximations of the data, the models were finally fit again using the true data, where the parameters found via the fitting routine with the spline approximations of the data were used as the initial parameters in the L-BFGS-B method.

A.6 Fit model values and parameters

Table A.5 shows the values of the parameters for the best fit models that resulted from model training. Fig A.5 shows the corresponding time series values representing each of the best fit models. The values include R_0, the compartment populations, and the predicted reported cases.

The best fit models were determined as those with the best log-likelihood score when fit over the training data, where the training data period was March 14 - May 31, 2020. See section A.8 for values from the robustness check.

Models were trained based on the standard SEIR model where:

$$\begin{aligned} S'(t) &= -\beta(t)\frac{S(t)I(t)}{N(t)} \\ E'(t) &= \beta(t)\frac{S(t)I(t)}{N(t)} - \sigma E(t) \\ I'(t) &= \sigma E(t) - \gamma I(t) \\ R'(t) &= \gamma I(t) \\ C'(t) &= rR'(t-d) \end{aligned}$$

and

$S(t) = N - E(t) - I(t) - R(t)$

$C(t)$ is cumulative case reports and accounts for reporting delay, d, and reporting rate, r.

The 3 models in this work differed based on how they defined transmission rate and the transition from S to E. See the Modeling section for details.

Model i: baseline model with constant transmission rate
Model ii: daily transmission a function of daily trips data
Model iii: daily transmission a function of daily trips and entrances data

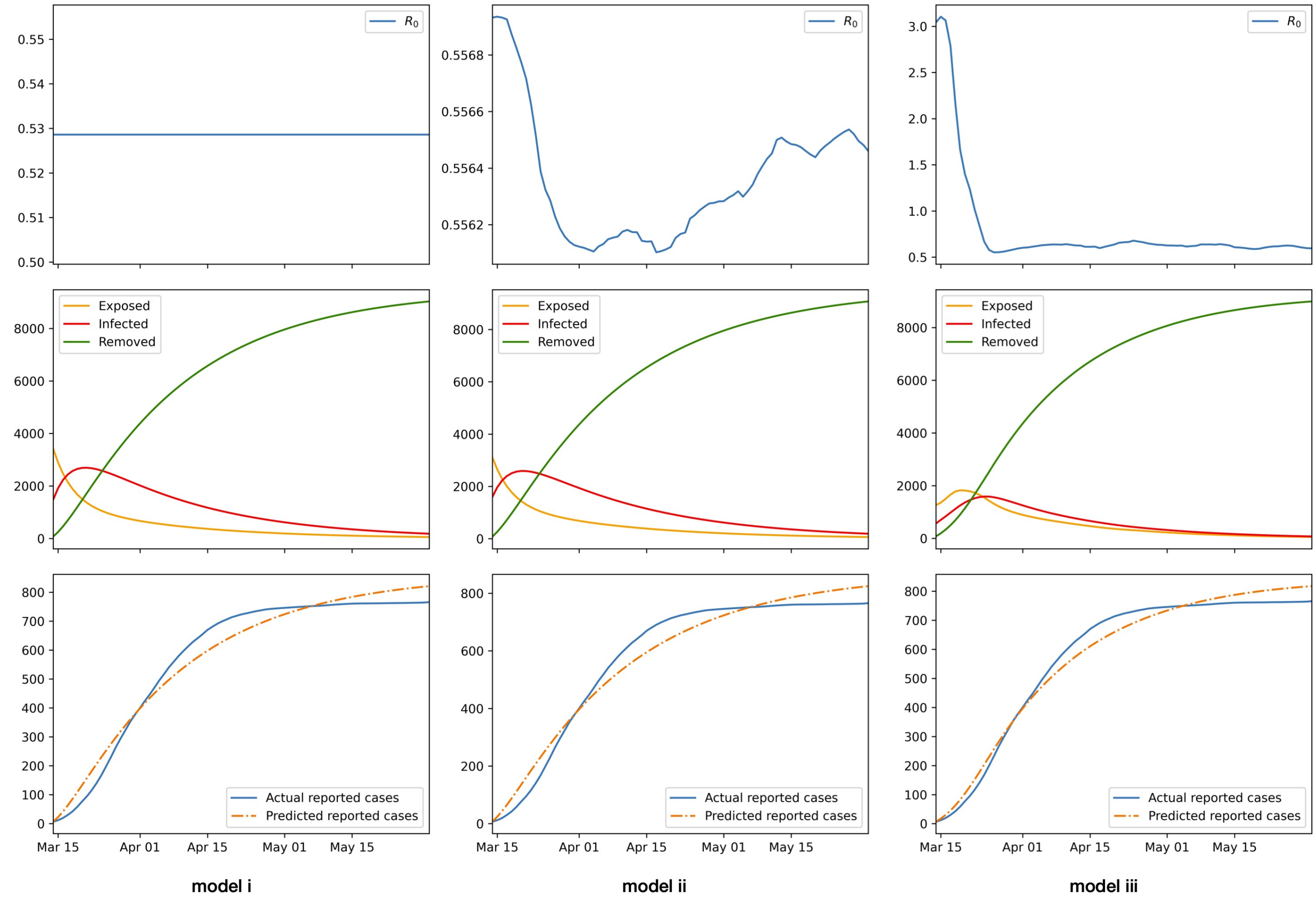

Fig A.5. Fit models. The time series values for (top) estimated R_0, (middle) estimated compartment populations, and (bottom) actual reported cases versus predicted reported cases. Plots are shown for each of the models: (left) model i is the baseline, dummy model with a constant transmission rate, (middle) model ii uses trips data, (right) model iii uses trips and entrances data.

parameter		model		
	description	i. constant β	ii. trips data	iii. trips & entrances data
N	Population. Set based on estimated population.	77,000	77,000	77,000
r	Reporting rate. Set based on serology and case reports data.	11^{-1}	11^{-1}	11^{-1}
d	Reporting delay. Set based on prior information.	7	7	7
σ	Latent rate. Set based on prior work.	5.2^{-1}	5.2^{-1}	5.2^{-1}
γ	Removal rate. Fit by model training.	0.10	0.10	0.18
$b0$	Transmission rate parameter. Fit by model training.	0.0529	0.0289	3.9126e-03
$b1$	Transmission rate parameter. Fit by model training.	-	0.027	7.69112e-02
$b2$	Transmission rate parameter. Fit by model training.	-	0.004	7.2082e-03
$b3$	Transmission rate parameter. Fit by model training.	-	-	2.6668e-06
$b4$	Transmission rate parameter. Fit by model training.	-	-	1.3168
E(0)	Initial exposed population. Fit by model training.	3408	3105	1261
I(0)	Initial infectious population. Fit by model training.	1488	1597	567
R(0)	Initial removed population. Set based on reported cases.	77	77	77
LL	Log-likelihood value for model fit over the training period	528.08	545.01	415.2

Table A.5. Parameters for fit models.

A.7 Time from exposure to reported case

The average delay in time from exposure to reported case in our model (i.e. the full transition through compartments E,I,R,C) is due to the average latent period, σ^{-1}, plus average infectious period, γ^{-1}, plus average reporting delay, d. σ^{-1} is set to 5.2 based on previous research [7], γ^{-1} is estimated via model training, and d is set to 7 based on related work [2, 8]. Reporting delays, d, can be due to the time it takes to seek a test, for the test to be processed, and then officially reported. Note that at the start of the pandemic in Andorra, tests were sent for processing to Spain, potentially adding extra time to reporting delays.

A study in Singapore from March 2020 estimated an average reporting delay of 6.4 days (95% CI 5.8, 6.9) [8]. A reporting delay of $d = 7$ was implicitly assumed by Arroyo-Marioli et al. [2]. They estimated time series values for the effective reproduction number for 124 countries across the world and validated their work by correlating their estimates of R_t to mobility data from the "COVID-19 Community Mobility Reports" collected by Google, where the lag between R_t and mobility was 14 days (2 weeks). They assumed an SIR model rather than an SEIR model, with time from exposure to removed, γ^{-1}, of 7 days, implying a reporting delay of 7 days $(14 - 7)$. We note that Arroyo-Marioli et al. produced time series estimates for R_t in Andorra. However their estimates are not comparable to the R_0 estimates in this work because (a) they did not correct for the reporting error that caused an influx of 78 additional cases June 1-10 [9], as was done in this work, and (b) their estimates were for the effective reproduction number versus the basic reproduction number. We also note that Google's "COVID-19 Community Mobility Reports" are not available for Andorra.

The best fit for the model estimated $\gamma^{-1} \sim 5.5$ days ($\gamma = 0.18$). Combined with $\sigma^{-1} = 5.2$, $d = 7$ results in a total estimated average delay from exposure to case report $\sim$17.7 days. This is consistent with previous work over a similar study period in Andorra that studied the correlation between mobility metrics and transmission rates over various lags and found the best correlations were with mobility metrics lagged by 18 days [10].

A.8 Robustness check

As a robustness check, we trained and tested all models over an additional set of training and testing periods that ended slightly earlier than those used for the main results. The training period for the robustness check was March 14 - May 14, 2020. The models were then tested on the period that directly followed this training period.

Table A.6 shows the comparison of MAPE values for each of the 3 models evaluated over the testing period. The model that uses the trips and entrances data consistently outperforms the other models.

	**MAPE**		
	**model**		
**forecasting weeks**	**i. constant β**	**ii. trips data**	**iii. trips & entrances data**
1	2.12	1.65	1.13
2	3.86	2.99	2.02
3	5.37	4.12	2.74
4	6.66	5.06	3.02
5	7.37	5.44	3.15
6	7.73	5.50	3.23
7	8.44	5.92	3.34
8	9.24	6.45	3.44
9	9.87	6.83	3.57
10	9.88	6.48	3.47

Table A.6. MAPE. MAPE values for the 3 models trained over the period used for a robustness check: March 14 - May 14, 2020.

Fig A.6 shows the resulting model time series values over the training period March 14 - May 14.

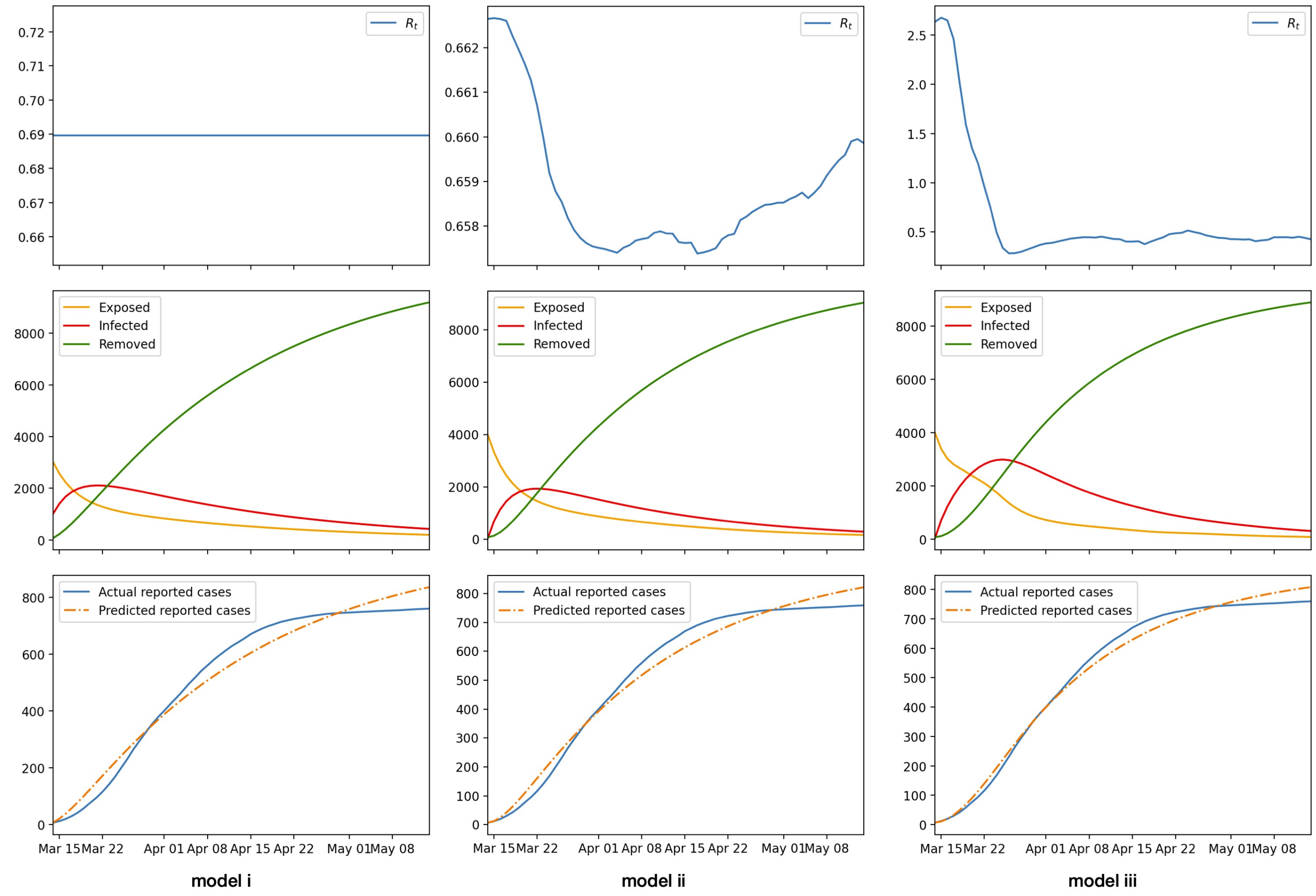

Fig A.6. Robustness check fit models. The time series values for the models fit for the robustness check. Values include (top) estimated R_0, (middle) estimated compartment populations, and (bottom) actual reported cases versus predicted reported cases. Plots are shown for each of the models: (left) model i is the baseline model and has a constant transmission rate, (middle) model ii uses trips data, (right) model iii uses trips and entrances data.

References

1. Departament-d'Estadística. Estimacions de població, gener 2020 and Estadística dels censos parroquials, gener 2020. Govern d'Andorra (Reports No A001 and A003). 2020; p. 1–11. https://www.estadistica.ad/serveiestudis/noticies/noticia5766fr.pdf.

2. Arroyo-Marioli F, Bullano F, Kucinskas S, Rondón-Moreno C. Tracking R of COVID-19: A new real-time estimation using the Kalman filter. PloS one. 2021;16(1):e0244474. https://doi.org/10.1371/journal.pone.0244474.

3. COVID-19 LMIC Reports: Frequently Asked Questions; 2020. MRC Centre for Global Infectious Disease Analysis, Imperial College London. `https://mrc-ide.github.io/global-lmic-reports/FAQ.html`.

4. Royo-Cebrecos C, Vilanova D, López J, Arroyo V, Pons M, Francisco G, et al. Mass SARS-CoV-2 serological screening, a population-based study in the Principality of Andorra. The Lancet Regional Health-Europe. 2021;5:100119.

5. Byrd RH, Lu P, Nocedal J, Zhu C. A limited memory algorithm for bound constrained optimization. SIAM Journal on scientific computing. 1995;16(5):1190–1208.

6. Virtanen P, Gommers R, Oliphant TE, Haberland M, Reddy T, Cournapeau D, et al. SciPy 1.0: Fundamental Algorithms for Scientific Computing in Python. Nature Methods. 2020;17:261–272. 10.1038/s41592-019-0686-2.

7. Li Q, Guan X, Wu P, Wang X, Zhou L, Tong Y, et al. Early transmission dynamics in Wuhan, China, of novel coronavirus–infected pneumonia. New England journal of medicine. 2020;.

8. Tariq A, Lee Y, Roosa K, Blumberg S, Yan P, Ma S, et al. Real-time monitoring the transmission potential of COVID-19 in Singapore, March 2020. BMC medicine. 2020;18:1–14.

9. Els tests d'anticossos permeten diagnosticar 78 positius de la COVID-19, que podrien haver contagiat unes 360 persones; 2020. Govern d'Andorra. `https://www.govern.ad/comunicats/item/11629-els-tests-d-anticossos-permeten-diagnosticar-78-positius-de-la-covid-19-que-podrien-haver-contagiat-unes-360-persones`.

10. Doorley R, Berke A, Noyman A, Alonso L, Ribo JF, Arroyo V, et al. Mobility and COVID-19 in Andorra: Country-scale analysis of high-resolution mobility patterns and infection spread. IEEE Journal of Biomedical and Health Informatics. 2021; p. 1–1. https://doi.org/10.1109/JBHI.2021.3121165.
