# Supplementary material for: Using mobile phone data to estimate dynamic population changes and improve the understanding of a pandemic: A case study in Andorra
Source: PLoS One. 2022 Apr 26;17(4):e0264860. doi: 10.1371/journal.pone.0264860 (PMC9041757; doi:10.1371/journal.pone.0264860)
Supplement: Attachment — Submitted filename: Response to Reviewers.pdf [file pone.0264860.s002.pdf]

Response to Reviewers

Manuscript number PONE-D-21-35256

Dear editor and reviewers,

We thank you for your thoughtful review and comments. The feedback has undoubtedly improved the original manuscript.

In the following letter we respond to each of your pieces of feedback separately.

Editor and journal requirements

> 1. Please ensure that your manuscript meets PLOS ONE's style requirements, including those for file naming.

Response
Please see that we have made necessary changes. We have reached out to the PLOS ONE staff and confirmed that our updated manuscript meets style requirements (we corresponded with Chloe Anderson, January 18).

> 2. Please provide additional details regarding participant consent.

Response
Please see the changes to our Methods section and Ethics statement that clarifies that the provided data was anonymized.

> 3. Please include a complete copy of PLOS' questionnaire on inclusivity in global research in your revised manuscript.

Response
Please see the uploaded file titled 'Questionnaire on inclusivity in global research' included in our submission file.

> 4. Please include your tables as part of your main manuscript and remove the individual files.

Response

Please note that we have updated the tables in our manuscript and confirmed with the PLOS ONE staff that our new manuscript meets journal guidelines for tables.

> 5. In your Data Availability statement, you have not specified where the minimal data set underlying the results described in your manuscript can be found.

Response
We have updated our data availability statement to be more informative. We are happy to note that both reviewers confirmed data availability. Reviewer #2 even ran the code and reproduced our results.

> 6. Please include a caption for figure 5.

Response
The original submission had a caption for this figure directly below Table 1, so no change has been made for this in the revised manuscript.

> 7. Please review your reference list to ensure that it is complete and correct.

Response
Thank you for pointing out that we should review our reference list. In general, we observed that some of our references were not easy to find by searching for them on the web. We added URL and DOI links to the references list, where appropriate, in order to address this. We have also added the references recommended by reviewer #1.

Reviewer 1

> The manuscript highlights a very important phenomenon on the role played by cases importation on local epidemics and how this can be best used in our current epidemic models to reproduce and understand the observed trends. The authors study the epidemic in Andorra which is indeed a very nice example of the role played by seeding thanks to the high ratio of external flows versus inhabitants.
> I think that the manuscript is well written and clear in the research purpose that it wants to address. The methodology and the data suit well this purpose.
>
> I think that the manuscript is suitable for acceptance after fulfilling a minor revision.

- “The value of 149 R0 can change over the course of an epidemic due to changes in human behavior and 150 NPIs; a goal of control efforts, such as those employed during the COVID-19 pandemic, 151 is to reduce R0 to bring an epidemic under control”
The authors make some confusion on the description of R0 (which does not change), probably confusing it with Rt.
Please check the definitions to avoid misconceptions and correct the paragraph.
https://royalsociety.org/-/media/policy/projects/set-c/set-covid-19-R-estimates.pdf

Response
We thank the reviewer for their positive words. We also thank the reviewer for highlighting this potential confusion in terminology. We have updated this section of the text to clarify our use of the term R0 and to distinguish this from Rt.

- line 212: “Model ii: transmission a function of trips data” → as

Response
We thank the reviewer for suggesting this grammatical change. We have made this change to both the Model ii and Model iii descriptions.

- line 244: It is not clear to me why C(t) should be proportional to the removals R with a delay in time. The cumulative reported cases should be reported with a certain delay after the individuals get infected, i.e. when they start being positive to tests, not when they get recovered or they isolate or die.

Response
We recognize that many other SEIR works have modeled the I compartment to represent the entire time an individual is infected, and then model R as "Removed" where Removed indicates they either recovered (are no longer infectious) or died.

However, given the guidelines and policies surrounding COVID-19, it became common for individuals to isolate themselves, and hence enter the (R) Removed compartment, as soon as they suspected themselves to be infected. For this reason, our modeling framework assumes that individuals transition from I to R as soon as they suspect they are infectious. Individuals may then seek a test, and the result of the test will be reported with some delay. We model C to represent the report of a positive test after that delay, d.

We thank the reviewer for pointing out that this was unclear. We have added additional text to the Model framework section to clarify how this assumption differs from many other SEIR models.

- line 315: “telecom data on mobility is provided by Andorra Telecom”, but then the authors say “Furthermore, telecoms data showed that 86% of entrances by foreign SIMs

were either Spanish or French, and when accounting for entrances by Andorran SIMs, 68% of all entrances were by Spanish or French SIMs".
So the mobility data includes also foreign countries SIM cards?

Response
We thank the reviewer for pointing out this should be clarified. In the original manuscript, we mentioned in the Andorra and COVID-19 section that Andorra Telecom data covers "...all mobile subscribers who spend any time in Andorra, whether they are Andorran nationals or have foreign SIM cards".

For clarity, we have also updated the Telecoms data and metrics section to say that Andorra Telecom data covers subscribers using foreign SIMs.

- on march 163h the govt of Andorra imposed a restriction to public activities and schools.
Your model is trained on this period until May 31, can this affect the calibration of your model to more general periods, like for example periods with no activity restrictions?

Response
We thank the reviewer for pointing out that we should better address this. In the Training and testing subsection we explain the data complications that cause us to use this period. The training period of March 14 - May 31 includes both severe restrictions as well as a gradual reopening of the economy. In the Model results subsection we explain that we also conduct a robustness check over a modified training and testing period, which does not include the Phase 3 reopening period, where results are summarized and then provided in further detail in the SI Appendix.

To further address the reviewer's point we have added text in the Model results section to comment on the impact of calibrating the model during the lockdown period. We have also added a comment in the discussion that notes how limiting our study to this specific period is a limitation to transferring our results to other contexts.

- Fig3 resolution is very poor and it is very difficult to understand as it is.

Response
We thank the reviewer for pointing this out. We have revised the figure and hope the reviewer finds it more informative.

- Fig4 shows a flat R0 for the trips-only model. However in Fig5 total trips seem to vary a lot during the period of observation. How come this scenario does not produce any variation of Rt with respect to trips (internal mixing)?

I understand that Andorra only counts for 77,000 inhabitants and that the external flow is very high with respect to the local population relatively to other countries, however I don't understand how a model based only on internal trips does not provide a varying reproductive number along the time of simulation.

Response
We note that while Fig 4 shows a flattened R0, this is due to the scale of the plot. There is variation which is fully shown in Fig A.5 in S1 Appendix. We call the reader's attention to these plots in the Model results subsection and particularly in the caption of Fig 4.

Even so, we agree with the reviewer that this flattened result with the trips data is surprising and reason as follows. During our modeling period, which followed Andorra's lockdown, we found that trips were not correlated with transmission rate (as shown in Fig 1). Due to the lack of predictive power of internal trips in the model, variation in these trips did not impact variation in the transmission rates. Since transmission rate was parameterized on trips only in this model, the modeled transmission rate was effectively almost constant. This counter-intuitive result demonstrated the need to incorporate more information - such as the entrances data - into the modeling of transmission rate.

As for how it could be the case that trips did not impact the modeled transmission rates, we have added text to address this following your comments below.

- line 432: only to get this clear in epidemiological terms, model 1 is a homogeneous mixing, model 2 is a model of one population in which transmission depends on local mixing only, model 3 accounts for local mixing and external seeding (importation and exportation)

- line 453: "More importantly, the model (ii) that used trips data to model transmission rates (without entrances data) had results similar to, and slightly worse than, the baseline model (i) which assumed a constant transmission rate. This is not surprising, as the data indicated trips were able to increase without impacting transmission rates"
I would say that this happens also because the model is calibrated in a lockdown period, hence internal mixing does not necessarily convert to spreading opportunities because of strong social distancing measures.

- line 454: "This is also shown in that the best fit for model (ii) had parameters that flattened the impact of the trips data, resulting in a nearly flat reproduction number, R0." indeed, the effect of the lockdown calibration, but of course using a fixed transmission parameter leads to this problem. The authors may need to address this model scenario limitation. (check gramatic of the first part of the sentence)

Response
We thank the reviewer for suggesting these clarifications. We have added text to the Model results section that describes the 3 models in the epidemiological terms that the reviewer

recommends. "In epidemiology, the 3 models may be considered as (i) a homogeneous mixing model, (ii) a model of one population in which transmission depends on local mixing only, and (iii) a model that accounts for local mixing and external seeding, where trips are a proxy for local mixing and entrances are a proxy for external seeding." We then add text to further interpret the results and address the fact that the lack of predictive power in the trips metric may be due to calibrating the model during a lockdown period.

> - Table 1: in the description of the error metric it would be better to state also the range of the values that this metric can reach. From 0 (perfect) to N (bad).

Response
In the revised manuscript, the following explanation has been added to the caption:
“The MAPE measures errors relative to the true values and can vary from 0 to infinity where 0 represents perfect agreement."

> - Fig.5, very interesting, why not plotting also the new cases time-series?

Response
We are glad that the reviewer found Figure 5 and the corresponding analysis interesting. Following the reviewer's suggestion, we have updated this figure to include a plot with the new cases timeseries.

> - Some recent and very related work on the effect on local epidemics of population changes and seeding caused by entrances is missing from the references, see for example: …

Response
We thank the reviewer for calling our attention to these related works. We have incorporated each of them into the Introduction section.

Reviewer 2

> The manuscript is very well written. The methods and results are described in clear, unambiguous language.
>
> The underlying data is presented in detail, including the mobility data results of the serological study. How mobility is measured and how the data is processed is described clearly, where the authors make use of data-processing practices commonly used in

literature (such as the stay detection and home detection). The serological study is properly discussed, including caveats stemming from the two waves of testing.

The data used in the study is made available online. The authors have also made software code used in the study available. I have downloaded the data and code and was able to replicate several results and figures used in the manuscript.

The SEIR model is set up, trained and evaluated appropriately. The two components of mobility are incorporated in a reasonable manner in the model. The use of three model scenarios is an appropriate way to test the influence of trips- and entrance-data on the prediction. I was honestly surprised by the extreme lack of predictive power of the model scenario using only trips data (model ii) and was very skeptical at first, but I could not find a flaw in the methodology. I do think that the entrances are such a good predictor as they are probably highly correlated with a host of other interventions that affect transmission rate, but the authors properly address this in the discussion. Altogether, I think the results, conclusions and their interpretations are sound.

Response
We thank the reviewer for both their skepticism and for thoroughly reviewing our methods and using our open source code and data to replicate results.

I would recommend the manuscript for publication as is.

I have only one minor remark which I would appreciate being addressed by the authors:

It is fair to say that Andorra is a very special case compared to other countries, due to its very small population and relatively large amount of cross-border traffic and temporary visitors. I would assume it has a much more "dynamic" population than most countries, which might mean that the results are not very transferable to other countries, where cross-border traffic and temporary visitors are of less importance. I think it would be appropriate to address this in the discussion. It would also be interesting to include comparative values for Andorra and other countries regarding how dynamic their populations are, if available (such as the fraction of cross border traffic among all traffic, the number of tourists relative to population, number of temporary workers, etc).

Response
We agree with the reviewer that our modeling approach, and hence results, are particularly well suited to a country like Andorra, where there is relatively high cross-border traffic compared to the stable population. We thank the reviewer for suggesting that we note this in the discussion and have added a paragraph describing this.
